# Renewable Hydrogen Production by Aqueous Phase Reforming of Pure/Refined Crude Glycerol over Ni/Al-Ca Catalysts

**DOI:** 10.3390/molecules28186695

**Published:** 2023-09-19

**Authors:** Raquel Raso, Eduardo Abad, Lucía García, Joaquín Ruiz, Miriam Oliva, Jesús Arauzo

**Affiliations:** Thermochemical Processes Group (GPT), Aragon Institute of Engineering Research (I3A), Universidad de Zaragoza, Mariano Esquillor S/N, 50018 Zaragoza, Spain; rroka@unizar.es (R.R.); 778205@unizar.es (E.A.); jruizp@unizar.es (J.R.); miroliva@unizar.es (M.O.); jarauzo@unizar.es (J.A.)

**Keywords:** hydrogen, pure/refined crude glycerol, Ni/Al-Ca catalysts, fixed bed reactor

## Abstract

Renewable hydrogen production by aqueous phase reforming (APR) over Ni/Al-Ca catalysts was studied using pure or refined crude glycerol as feedstock. The APR was carried out in a fixed bed reactor at 238 °C, 37 absolute bar for 3 h, using a solution of 5 wt.% of glycerol, obtaining gas and liquid products. The catalysts were prepared by the co-precipitation method, calcined at different temperatures, and characterized before and after their use by several techniques (XRD, ICP-OES, H_2_-TPR, NH_3_-TPD, CO_2_-TPD, FESEM, and N_2_-physisorption). Increasing the calcination temperature and adding Ca decreased the surface area from 256 to 188 m^2^/g, and its value after the APR changed depending on the feedstock used. The properties of the acid and basic sites of the catalysts influenced the H_2_ yield also depending on the feed used. The Ni crystallite was between 6 and 20 nm. In general, the incorporation of Ca into Ni-based catalysts and the increase of the calcination temperature improved H_2_ production, obtaining 188 mg H_2_/mol C fed during the APR of refined crude glycerol over Ni/AlCa-675 catalyst, which was calcined at 675 °C. This is a promising result from the point of view of enhancing the economic viability of biodiesel.

## 1. Introduction

The past twenty years have seen annual increases in world energy demand mainly due to population growth, together with the requirements of industry and the development of society in general. Most energy comes from the combustion of fossil fuels, such as petroleum oil, coal, and natural gas [1]. However, the consumption of fossil fuels causes environmental problems, such as the greenhouse effect and pollution emissions. Hence, developing and utilizing renewable energies are essential for addressing environmental pollution and the energy crisis [2]. The International Energy Agency (IEA) estimates energy consumption growth from sustainable resources will reach 53% by 2030 [3]. Hence, much research has been devoted to seeking alternatives to reduce the dependence on petroleum and increase the use of renewable energy, such as biodiesel [1,4].

Biodiesel is mainly produced by the catalytic transesterification of triglycerides with alcohol, thereby obtaining glycerol (10% by weight) as a by-product [1]. As the biodiesel industry rapidly expands, a surplus of glycerol is being created, which could negatively affect the biodiesel economy. Therefore, it is fundamental to find efficient alternatives to valorize glycerol [5,6]. In this context, H_2_ production from glycerol is a promising method to reduce environmental problems, contributing to the expansion and economic viability of biorefinery [7].

H_2_ is the simplest and the most abundant element in the universe, but it is rarely present as gas on Earth [8]. It is considered a clean energy, which only emits water after its combustion. It can be used in many technologies, such as fuel cells for electricity production [9,10,11]. There are many routes to produce H_2_ from glycerol, such as auto-thermal reforming (ATR, [10,12]), steam reforming (SR, [13,14]), and aqueous phase reforming (APR, [15]).

Among these processes, APR is kinetically and thermodynamically feasible for producing H_2_ with a low CO concentration [16]. APR was first investigated by the group of Dumesic in 2002 [15]. It is a catalytic process that operates at mild temperatures (200–270 °C) and moderate pressures (25–50 bar) [17]. APR is also energy-efficient because glycerol and water are not vaporized [18], and separating the APR products is easy; hence, it has some advantages over SR [17]. In addition, it is the only process in which the reforming is carried out in the liquid phase [19].

The ideal APR of glycerol yields seven moles of H_2_ and three moles of CO_2_ per mole of glycerol, combining the decomposition of glycerol and the water–gas shift [16,18] (Equations (1)–(3)).

C-C cleavage leading to CO and H_2_:

(1) $$C_{3}H_{8}O_{3}\;(l)\;\to \;\;\;3CO\;(g)+4H_{2}\;(g)\;\;\;\;\;\;\Delta {H{}^{\circ}}_{25C}=\;+338.0\;kJ/\mathrm{mol}$$

Water–gas shift (WGS):

(2) $$\mathrm{CO}\;(g)+H_{2}O\;(l)\;⇄\;\;\;CO_{2}\;(g)+H_{2}\;(g)\;\;\;\;\;\;\Delta {H{}^{\circ}}_{25C}=\;+2.8\;kJ/\mathrm{mol}$$

The overall reaction stoichiometry (ideal APR of glycerol):

(3) $$C_{3}H_{8}O_{3}\;(l)+3H_{2}O\;(l)\;\to \;\;\;3CO_{2}\;(g)+7H_{2}\;(g)\;\;\;\;\;\;\Delta {H{}^{\circ}}_{25C}=\;+346.5\;kJ/\mathrm{mol}$$

A suitable catalyst for the APR process should be active mainly in the C-C bond scission and the water–gas shift reaction. Pt-based catalysts are often used due to their ability to break C-C bonds and their efficiency for SR/WGS reactions [16,20]. Many studies about H_2_ production by the APR of glycerol over Pt-based catalysts have been reported [21,22,23,24,25].

Menezes et al. [26] studied the influence of different oxide supports (Al_2_O_3_, MgO, ZrO_2_, and CeO_2_) on Pt-based catalysts for APR of glycerol using a batch reactor. They found that Pt/MgO and Pt/ZrO_2_ showed the best activity for H_2_ production with low hydrocarbon formation due to their nature as basic oxides, thereby inhibiting methane formation and favoring the WGS reaction.

However, the high cost and the limited availability, in particular, of noble metals (to produce H_2_ on an industrial scale) have led to studies aimed towards developing cheap catalysts, such as Ni-based catalysts [27]. Furthermore, Ni-based catalysts have attracted the attention of many authors [17,27,28,29,30] due to their good intrinsic C-C cleavage, but their inhibition ability is weak for methane reactions [18,31] (Equations (4)–(6)). 

Methanation reactions:

(4) $${\mathrm{CO}}_{2}\;(g)+4H_{2}\;(g)\;⇄\;CH_{4}\;(g)+2H_{2}O(1)\;\;\;\;\;\;\Delta {H{}^{\circ}}_{25\;{}^{\circ}C}=\;-253.0\;kJ/\mathrm{mol}$$

(5) $$\mathrm{CO}\;(g)+3H_{2}\;(g)\;⇄\;CH_{4}\;(g)+H_{2}O\;(1)\;\;\;\;\;\;\Delta {H{}^{\circ}}_{25\;{}^{\circ}C}=\;-250.2\;kJ/\mathrm{mol}$$

(6) $$2\mathrm{CO}\;(g)+2H_{2}\;(g)\;⇄\;CH_{4}\;(g)+CO_{2}\;(g)\;\;\;\;\;\;\Delta {H{}^{\circ}}_{25\;{}^{\circ}C}=\;-247.3\;kJ/\mathrm{mol}$$

The production of H_2_ and CO_2_ by APR at low temperatures may be accompanied by methanation/Fischer–Tropsch reactions, which could generate methane and other alkanes, reducing the selectivity to H_2_ [32].

García et al. [33] and Remón et al. [34] analyzed the influence of the operating variables on the APR of glycerol over Ni co-precipitated catalysts using a continuous fixed bed reactor. Remón et al. [34] concluded that the best operating conditions for obtaining a high gas yield were 238 °C, 39 bar, and high W/m_glycerol_ ratio (38 g_catalyst_·min/g_glycerol_). García et al. [33] found that a low glycerol content in the solution increased the H_2_ yield. Moreover, the H_2_ content in the gas decreased while the CH_4_ content increased with rising pressure [33]. As such, some favorable operating conditions to obtain a high H_2_ yield can be: 238 °C, 37 bar, 5 wt.% of glycerol, and 40 g_catalyst_·min/g_glycerol_.

Ni-based catalysts can be enhanced and stabilized by employing certain supports and metals, thereby obtaining a high yield of hydrogen in APR and inhibiting the deactivation of the catalysts due to coke formation [31]. Alumina as a support has attracted considerable interest due to its high surface area, which enhances metal dispersion. However, it is known that alumina supports are susceptible to suffering deactivation due to metal particle sintering and carbon deposition, issues that negatively affect their long-term stability [35,36,37,38]. The formation of coke deposits has been associated with cracking, polymerization, and dehydration reactions, which occur on the acid sites of alumina, while sintering has been related to a transition of alumina to a crystalline phase during the reaction [35]. Therefore, many authors have reported using Cu, Zn, La, Br, and Co as structural modifiers of Ni/Al_2_O_3_, effectively promoting the APR of glycerol. In addition, basic oxides, such as La_2_O_3_, CeO_2_, MgO, or CaO, have been used to modify or neutralize the acid alumina [17]. Hence, the design of a stable support material with high selectivity to H_2_ is key for the viability of the APR process. Shabaker et al. [39] reported that adding Sn to Ni favored the reduction of methane by reducing the C-O fracture and the improvement of hydrogen production by increasing the C-C fracture. Iriondo et al. [40] investigated the APR of glycerol over monometallic (Ni or Pt) and bimetallic (PtNi) catalysts supported on γ-Al_2_O_3_ and La_2_O_3_-modified γ-Al_2_O_3_ using a fixed bed reactor. They observed that PtNi catalysts were the most active, while Ni catalysts suffered an increasing deactivation with the temperature. In addition, the presence of La_2_O_3_ in the catalysts improved the H_2_ production. 

Morales-Marín et al. [17] studied the effect of promoters (Ce or Mg) in the NiAl_2_O_4_ spinel-derived catalysts for the APR of glycerol using a fixed bed reactor. They found that adding the promoters slightly increased the selectivity to hydrogen, diminishing the CO/CO_2_ and CO/H_2_ ratio in the gas phase products.

With this background, the present work studied the influence of adding Ca to the Ni/Al catalyst during the APR of glycerol.

CaO has been employed as a promoter of Ni/Al_2_O_3_ catalysts because of its basicity, low cost, and wide availability. It not only neutralizes the acid sites on alumina but also favors H_2_O adsorption and -OH mobility, thus accelerating carbon oxidation and inhibiting coke deposition [13,41]. Moreover, CaO can adsorb CO_2_ during the reforming system [41]. 

Compared with pure glycerol, crude glycerol includes many impurities, such as soap, alkali, ester, salts, and non-glycerin organic compounds, which will affect the APR for H_2_ production. Therefore, it is necessary to understand the influence of the impurities in crude glycerol during APR for H_2_ production [2]. A few studies have used crude glycerol during APR [31,34,42]. Boga et al. [43] studied the effect of the impurities (soap, methanol, and ester) in crude glycerol (containing 6.85 wt.% glycerol, 1.62 wt.% soaps, 1.55 wt.% methanol, and 0.07 wt.% ester) during APR over Pt-based catalysts at 29 bar and 225 °C, obtaining less activity with the crude glycerol compared with pure glycerol (6.85 wt.%). Lenhert and Claus [44] analyzed the APR of pure and crude glycerol using several Pt-based catalysts at 250 °C and 20 bar. They observed that the presence of NaCl in crude glycerol affected H_2_ production and caused more significant catalyst deactivation than pure glycerol. Other authors [2,45,46] have investigated the effect of impurities, such as methanol, acetic acid, KOH, sulfuric acid, NaOH, and phosphoric acid, in pure glycerol during APR over Ni-based catalysts. They found that methanol and acetic acid impurities negatively affected the APR, causing a decrease in the conversion to gases and glycerol conversion. At the same time, the KOH increased the glycerol conversion and enhanced H_2_ production. They also found that Ni leaching increased under acidic conditions. In addition, Wu et al. [2] reported that including CaO in the bed can increase the H_2_ conversion and production rate in the APR of a glycerol solution containing impurities.

Taking into consideration the possible presence of three impurities (methanol, acetic acid, and KOH) in crude glycerol [34,42] and the effect of CaO on the H_2_ production studied by other authors [2,13,31], this work investigated the impact of adding Ca as a promoter in APR using crude glycerol. KOH is usually employed as a homogeneous catalyst, while methanol is used to react with triglycerides in biodiesel production. Acetic acid helps remove the soaps in crude glycerol during its purification step [4,34].

This study has two main innovations in the context of catalyst development for glycerol valorization. The first one is the preparation and characterization of Ni/Al coprecipitated catalysts with Ca using NH_3_-TPD and CO_2_-TPD, among other techniques. The metals in these catalysts (Ni, Al, and Ca) are not expensive and have high availability. The second one is the use of these catalysts in the APR of glycerol, including pure and refined crude glycerol. This research is relevant for the purpose of H_2_ production using as the feed an industrial by-product, glycerol, employing low-cost catalysts.

To the best of our knowledge, this is the first study on the effect of Ca on Ni/Al catalysts during APR using pure and refined crude glycerol with the goal of increasing the H_2_ yield. 

The Ni/Al-Ca catalysts were prepared by the co-precipitation method, changing the molar ratio of Ca/Al from 0 to 7.5%. After calcination, the phases presented in the catalysts were CaO, Al_2_O_3_, NiAl_2_O_4_, and NiO. For simplicity, the catalysts were named Ni/Al-Ca. In addition, the influence of the calcination temperature was analyzed, characterizing the fresh and used catalysts by several techniques, such as hydrogen temperature-programmed reduction (H_2_-TPR), inductively coupled plasma optical emission spectrometry (ICP-OES), field emission scanning electron microscopy (FESEM), X-ray diffraction (XRD), N_2_-physisorption, temperature-programmed desorption of ammonia (NH_3_-TPD), and carbon dioxide (CO_2_-TPD).

## 2. Results

In the present work, two types of glycerol were used during the aqueous phase reforming: (1) pure glycerol as a chemical reagent, and (2) refined crude glycerol obtained from biodiesel production. 

### 2.1. Physicochemical Characteristics of the Fresh Catalysts

#### 2.1.1. Composition and Textural Properties

The theoretical value of the molar ratio (expressed as %) of the Ca/Al in the catalyst with Ca was 7.5%. Table 1 shows the Ni, Al, and Ca contents determined by inductively coupled plasma optical emission spectrometry (ICP-OES). The metal content was in good agreement with the theoretical value for all catalysts except for the Ni/AlCa-500 catalyst (sample with Ca and calcined at 500 °C). This error could be related to the agitation rate and the velocity rate of the addition of the precipitate agent until the required final pH, among others.

**Figure 1:**
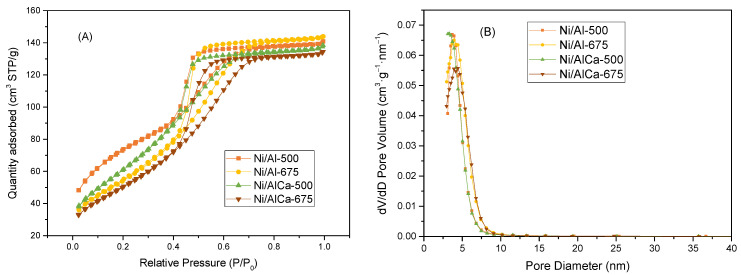
N_2_ adsorption–desorption isotherms (**A**) and pore size distribution (**B**) of the calcined catalysts.

All catalysts presented a type IV isotherm with a hysteresis loop (type H2), according to the International Union of Pure and Applied Chemistry (IUPAC) classification [48], which is typical of mesoporous materials, as shown in Figure 1A. The H2-type hysteresis loop indicates particles with an irregular structure and a non-uniform shape and size [13]. 

The hysteresis loop shifted to higher relative pressure (P/P_0_) with an increase in the calcination temperature, which suggests that framework pores were gradually changed into textural pores because of sintering, as reported by Yu et al. [11]. The pore size distribution was calculated using the Barrett–Joyner–Halenda (BJH) adsorption method, shown in Figure 1B. The pore diameter of the samples was in the range of 3–4 nm (Table 2), corresponding to the mesopores structure [48].

Table 2 displays the textural properties of the calcined catalysts. The surface area was obtained using the Brunauer–Emmett–Teller (BET) method. The addition of Ca to the Ni/Al catalysts decreased the specific surface area from 256 to 232 m^2^/g for the catalysts calcined at 500 °C and from 203 to 188 m^2^/g for the catalysts calcined at 675 °C. This could be due to the small specific surface area of CaO, which would lead to such decreases [49]. Other authors have found a similar trend [49,50,51].

In addition, it was observed that the increase in the calcination temperature also diminished the specific surface area from 256 to 203 m^2^/g for the Ni/Al catalyst and from 232 to 188 m^2^/g for the Ni/AlCa catalyst, the same trend as reported by other authors [11,51,52]. 

#### 2.1.2. Crystalline Structure

**Figure 2:**
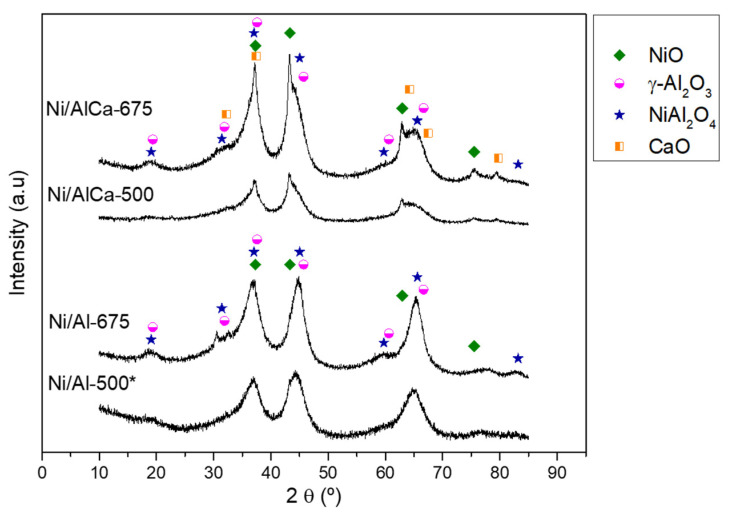
XRD patterns of the calcined catalysts. * Raso et al. [47].

The crystalline phases of the catalysts were determined by X-ray diffraction (XRD). The XRD patterns of the calcined samples are shown in Figure 2. 

The patterns show an amorphous structure, which was more crystalline with the increase in the calcination temperature. For this reason, it was difficult to identify the characteristic phases. All catalysts presented the γ-Al_2_O_3_ (JCPDS 00-050-0741) and NiAl_2_O_4_ (JCPDS, 00-010-0339) and NiO (JCPDS, 00-047-1049) phases in their structure. In addition, it was observed that the Ni/AlCa (Ni/AlCa-500 and Ni/AlCa-675) catalysts showed an additional peak corresponding to the CaO (JCPDS, 00-037-1497) phase. 

The calculation of the NiO crystalline size was not possible for all of the catalysts due to the overlap between nickel oxide and the other phases. The results showed that the crystallinity increased with an increase in the calcination temperature, demonstrating the agglomeration of particles at higher temperatures [13]. In addition, adding Ca favored the clarity of the peak characteristic of NiO phases at 2θ = 43, 63, and 75°, especially in the Ni/AlCa-675 catalyst. There were no peaks corresponding to the interaction between CaO and Al_2_O_3_ to form calcium aluminates (Ca_4_Al_6_O_13_, CaAl_2_O_4_, or Ca_12_Al_14_O_33_) due to the weak Ca-Al interaction during the catalyst preparation [11].

#### 2.1.3. Reducibility

**Figure 3:**
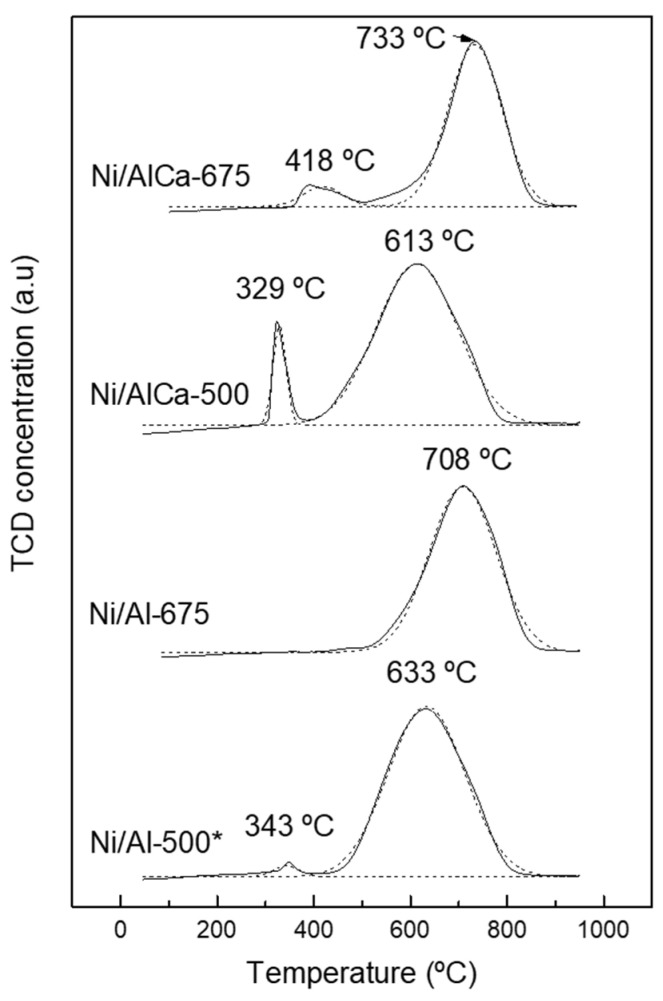
H_2_-TPR profiles of the calcined catalysts. * Raso et al. [47].

The reducibility of the fresh samples was measured by hydrogen temperature-programmed reduction (H_2_-TPR). Figure 3 displays the H_2_-TPR profiles of the calcined samples. All catalysts showed two peaks except the Ni/Al-675 catalyst, which presented only one peak. The first peaks (329–418 °C) are attributed to the reduction of the NiO phase with less interaction with the support (α-NiO). Meanwhile, the second peaks (613–733 °C) are ascribed to the reduction of the NiO with strong interaction with the support (γ-NiO, NiAl_2_O_4_). Generally, the peaks at low temperatures (200–440 °C) are assigned to the reduction of α-NiO, while at the high temperatures (550–770 °C), they are attributed to the reduction of γ-NiO [53]. 

The ratio of the different phases was calculated, as displayed in Table 3. The addition of Ca favored the increase in the proportion of α-NiO, whereas γ-NiO decreased, probably because the Ni in the spinel phase is replaced by the NiO phase, as reported by Medrano et al. [50]. They found that the increase in the Ca/Al molar ratio increased the first peak but decreased the second one in H_2_ consumption. Elias et al. [54] also found the same trend and suggested that it could be because of competition between the calcium and nickel to interact with the Al_2_O_3_, causing the formation of nickel species with weak Al_2_O_3_ interaction, as also reported by Dias et al. [55]. The H_2_-TPR results are in accordance with the XRD patterns due to the increment of the intensity of the NiO phase with Ni/AlCa catalysts.

Furthermore, the reduction temperature peaks corresponding to the spinel moved towards higher values with the increase in the calcination temperature from 633 to 708 °C and from 613 to 733 °C for the Ni/Al and Ni/AlCa catalysts, respectively. Raso et al. [52] observed an increase in the reduction temperature peaks of γ-NiO/FeAl_2_O_4_ from 568 to 802 °C with the rise of the calcination temperature from 500 to 750 °C.

The total H_2_ consumption for the catalysts was an average of 4.5 mmol H_2_/g. The percentage of Ni_red_ increased with the addition of Ca, obtaining 100% with the Ni/AlCa-500 catalyst.

#### 2.1.4. Acidity and Basicity

The acidity and basicity of the fresh catalysts were analyzed by the temperature-programmed desorption of ammonia (NH_3_-TPD) and carbon dioxide (CO_2_-TPD), respectively.

**Figure 4:**
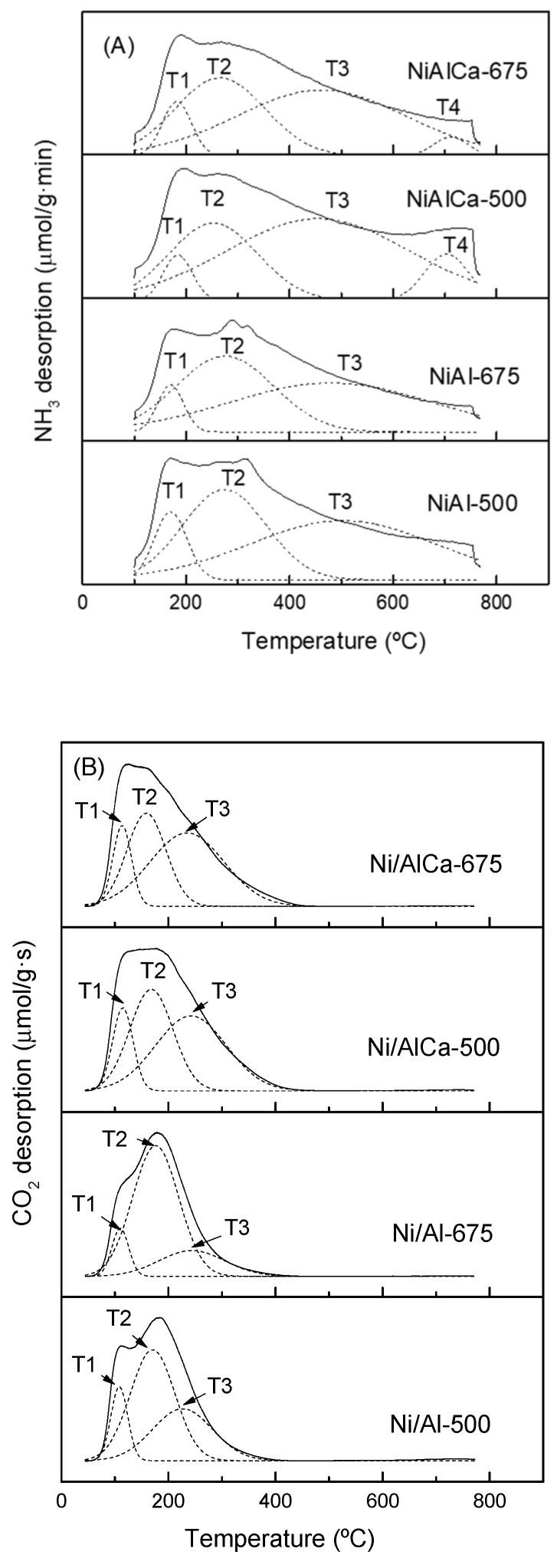
NH_3_-TPD (**A**) and CO_2_-TPD (**B**) profiles of the reduced catalysts.

The NH_3_-TPD profiles of the reduced catalysts are shown in Figure 4A. Before the TPD analysis, the samples were reduced at the same temperature as that used to activate them before the APR reaction: 600 °C for the catalysts calcined at 500 °C (Ni/Al-500 and Ni/Al-Ca-500) and 700 °C for the samples calcined at 675 °C (Ni/Al-675 and Ni/Al-Ca-675). 

The Ni/Al catalysts presented three regions characteristic of the strength of the acid sites, while the Ni/AlCa catalysts showed four zones. Hence, the different regions were classified according to the maxima peak and reported by other authors [47,56,57] as weak (170–184 °C), low-moderate (253–275 °C), strong (455–503 °C), and very strong (703–711 °C).

It was observed that the incorporation of Ca into the catalyst decreased the value of the maximum temperature (T2 and T3) and the proportion of the low-moderate (F2) acid sites. This proportion changed from 37.1 to 28.1% and from 41.9 to 20.4% for the catalysts calcined at 500 °C and 675 °C, respectively (Table 4). According to a report by Elias et al. [54], when alkali is inserted into alumina, it acts as a poison to the alumina Lewis acid sites. Perhaps Ca has a similar effect.

In addition, the total acidity, expressed as μmol NH_3_/g_cat,_ decreased when adding Ca and raising the calcination temperature. The decrease in the total acidity with the presence of Ca was more significant for the catalysts calcined at 500 °C. Nevertheless, the total acidity, expressed as μmol NH_3_/m^2^ (density of acid sites), diminished only when adding Ca and increasing the calcination temperature for the Ni/Al catalysts. For the Ni/AlCa catalysts, an increase from 2.29 to 2.62 μmol NH_3_/m^2^ of the density of acid sites was observed with a rising calcination temperature. This was due to the larger BET surface area of the Ni/AlCa-500 than the Ni/AlCa-675 in proportion to the total acidity value. 

Figure 4B displays the CO_2_-TPD profiles of the reduced catalysts. Before the TPD analysis, the samples were reduced at the same temperature as that used to activate them before the APR reaction under the operating conditions cited above for the NH_3_-TPD analysis. CO_2_ is an acidic gas that was adsorbed on some basic sites of the catalysts under reaction conditions [58].

All catalysts showed three regions corresponding to the strength of the basic surface sites, which depend on the different desorption temperatures of the CO_2_ [16]. According to Guo et al. [16], the first (T1, 108–115 °C) and second peaks (T2, 159–176 °C) are associated with the occurrence of weak basic surface sites, while the third (T3, 226–243 °C) is considered a different distribution of surface centers with relatively strong basic sites.

As reported by Boukha et al. [59], the desorption at low temperatures (40–150 °C) is related to the decomposition of bicarbonate species, and at medium temperatures (150–350 °C), it is attributed to the decomposition of bidentate carbonate species.

Adding Ca increased the proportion of the first (F1) and third (F3) peaks but decreased that of the second peak (F2) (Table 5). Sabokmalek [13] et al. reported an increase in the strong basic sites with a decrease in the Al/Ca ratio. García-Bordejé et al. [58] observed that incorporating alkaline metals (K, Ba, and Na) into Ni/Al and Ru/Al catalysts increases the amount of weak and strong basic sites. The presence of alkali produces a rise in the number and strength of the O^2−^ basic sites on the catalyst surface [54]. For monometallic catalysts, CO_2_ could be adsorbed on Lewis basic sites of the metal (Ru or Ni) and Brønsted basic hydroxyl groups of the Al_2_O_3_ support [58].

In addition, the total basicity, expressed as μmol CO_2_/g_cat_, increased with increasing the Ca; for example, from 81.44 to 156.17 μmol CO_2_/g_cat_ for the Ni/Al-500 and Ni/AlCa-500 catalysts, respectively. However, it diminished when the calcination temperature was raised; for example, from 81.44 (Ni/Al-500) to 73.56 μmol CO_2_/g_cat_ (Ni/Al-675) in the case of the Ni/Al catalysts. Goma et al. [60] reported an increase in basicity after adding Ca to Ni-based catalysts (Ni/CSZ catalysts, where CSZ is Calcia-Stabilized Zirconia). Barzegari et al. [61] observed a reduction in basic sites when the calcination temperature increased. Nevertheless, for the Ni/Al catalysts, a rise from 0.32 to 0.46 μmol CO_2_/m^2^ in the density of basic sites was observed with an increasing calcination temperature due to the BET surface area of the Ni/Al-500 being larger than that of the Ni/Al-675 in proportion to the total basicity value.

#### 2.1.5. Morphology

**Figure 5:**
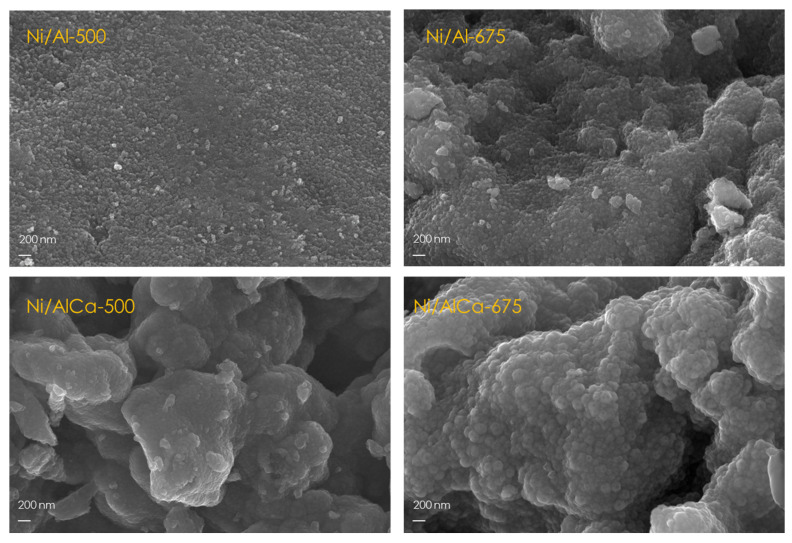
FESEM images of the calcined catalysts.

The fresh catalyst morphology was analyzed by field emission scanning electron microscopy (FESEM). Figure 5 shows the FESEM images of the calcined catalysts. It was observed that by increasing the calcination temperature and adding Ca, the Ni/Al catalyst morphology was slightly changed. An increase in calcination temperature enhanced the particle size due to the agglomeration occurrence in a hot environment, as reported by Sabokmalek et al. [13].

### 2.2. Results from Pure Glycerol

#### 2.2.1. Catalytic Performance

**Figure 6:**
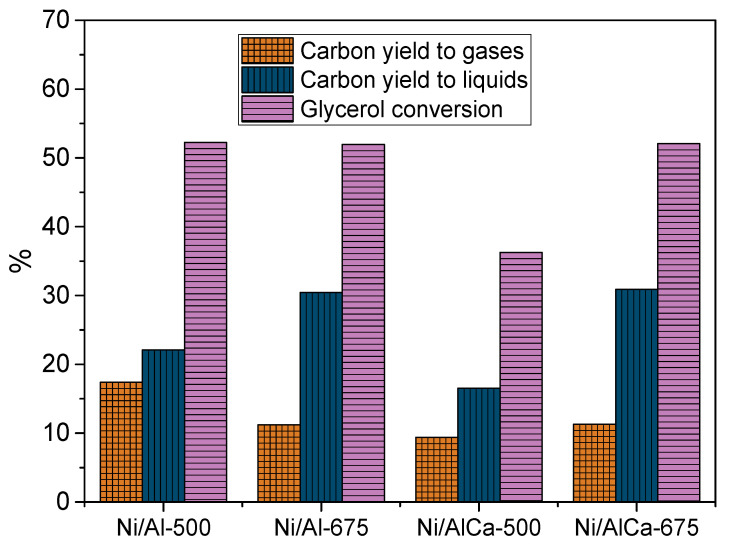
The catalytic activity of the catalysts during the APR of pure glycerol.

Figure 6 shows the catalytic activity of the catalysts during the APR of glycerol. It was observed that the glycerol conversion was around 52% for all of them, except for the Ni/AlCa-500, maybe because of the low amount of Ni content in that catalyst and its smaller pore diameter. According to the ICP-OES analysis, the Ni/AlCa-500 was the worst synthesized, with around 7% of relative error. Adding Ca to the catalysts calcined at 675 °C did not affect the glycerol conversion or carbon yield to products (liquid and gas). Increasing the calcination temperature for the catalysts without Ca favored the production of the carbon yield to liquid but reduced the carbon yield to gas. The distribution of the carbon selectivity to liquid products and the ratios of 1,2-PDO/acetol and (ethanol + ethylene glycol)/(acetol + 1,2-PDO) were very similar for the different catalysts studied, demonstrating that these phenomena did not depend on the catalyst (Figure S1). 

The purpose of this work was to increase the carbon yield to gases and, principally, the hydrogen yield. To achieve this, the APR of glycerol was carried out at 238 °C and 37 bar for 3 h, using a solution of 5 wt.% of glycerol in milli-Q water, which was pumped into the reactor at 1 mL/min. A comparison of the results obtained in this work with those reported by Raso et al. [47], who carried out the catalytic test at 227 °C and 34 bar, using the same Ni/Al-500 catalyst but 10% of glycerol, revealed an increase in the carbon yield to gases from 5.1% [47] to 17.4% and in the glycerol conversion from 24.7% [47] to 52.3%. These results corroborate the significant influence of the operating conditions on the products obtained.

Increasing the calcination temperature for catalysts without Ca and then adding Ca favored an increase in the H_2_ content in the product gas. It also enhanced the ratio of H_2_/CO_2_ in the APR of glycerol, as shown in Table 6. The stoichiometric H_2_/CO_2_ ratio should be 2.3 in an ideal glycerol APR reaction [17]. The samples showed an H_2_/CO_2_ ratio of around 0.34 to 0.73, suggesting that some hydrogen was consumed in parallel reactions; for example, the hydrogenation of acetol to 1,2-propanediol.

**Figure 7:**
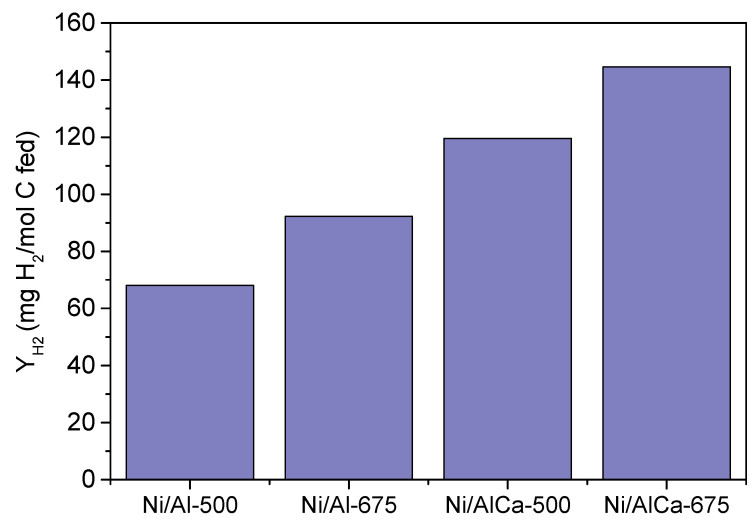
Hydrogen yield (Y_H2_) of all the catalysts during the APR of pure glycerol.

It was also observed that the hydrogen yield, expressed as mg H_2_/mol C fed, was increased by adding Ca and increasing the calcination temperature, as shown in Figure 7. Therefore, these two parameters positively influence hydrogen production during the APR of glycerol. Moreover, no carbon monoxide was detected, which is beneficial for H_2_ application in PEM (proton exchange membrane) fuels.

Overall, the catalysts showed amphoteric surface properties. The catalysts with Ca (Ni/AlCa-500 and Ni/AlCa-675) showed a low density of acid sites (i.e., inhibition of dehydration reactions) and a high density of basic sites (i.e., promotion of WGS reactions) related to enhanced hydrogen selectivity [17]. The H_2_ yield follows the order Ni/AlCa-675 > Ni/AlCa-500 > Ni/Al-675 > Ni/Al-500, which is opposite to the total acidity expressed as μmol NH_3_/g_cat_. This fact could indicate a relationship between low acidity and H_2_ yield.

#### 2.2.2. Catalyst Characterization

Table 7 shows the textural properties of the spent catalysts. The specific surface area and pore volume of the catalysts without Ca decreased after use. However, the pore diameter increased for the Ni/Al-675 catalyst from 3.9 to 6.8 nm, with a decrease in the surface area of around 33%. Adding Ca increased the specific area of the catalysts after use, diminishing the pore volume and diameter. 

**Figure 8:**
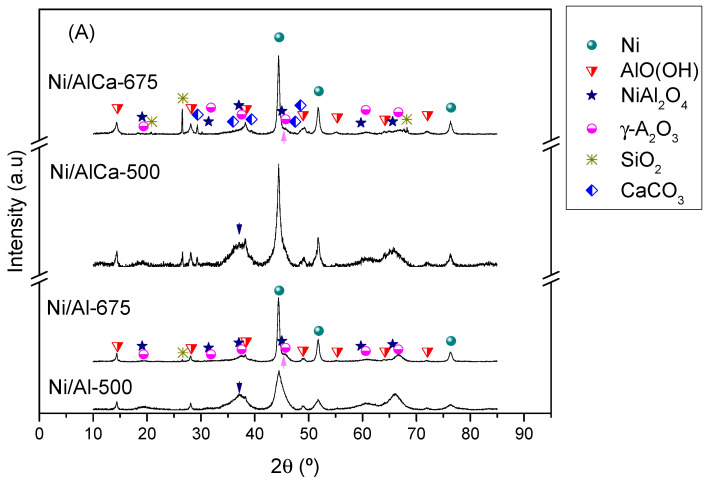

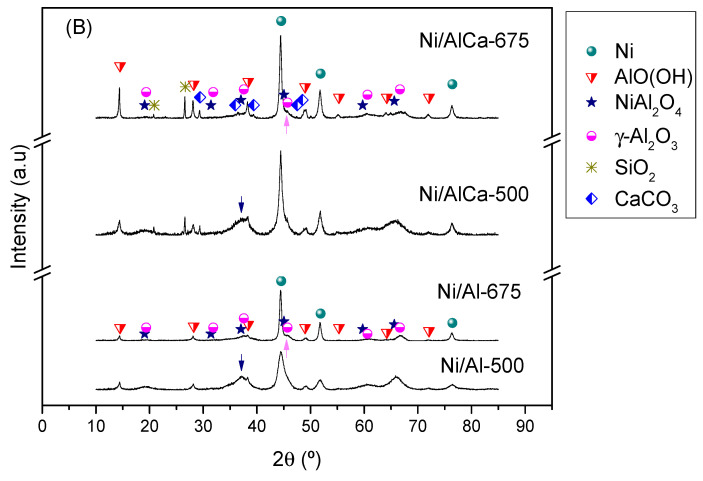
XRD patterns of the spent catalysts using different glycerol as feed: (**A**) pure glycerol and (**B**) refined crude glycerol. Blue arrow: NiAl_2_O_4_ at 2θ = 37.0°; pink arrow: γ-Al_2_O_3_ at 2θ = 45.7°.

The XRD patterns of the spent catalysts were analyzed in order to determine the crystalline phases after use, as shown in Figure 8A. The XRD patterns of the reduced samples were not analyzed because Raso et al. [47] reported no significant differences between the reduced and spent catalysts, and only the appearance of an additional peak characteristic of the boehmite in the used catalysts.

All the catalysts had phases in their structure corresponding to Ni (JCPDS 00-004-0850), γ-Al_2_O_3_, and NiAl_2_O_4_ and boehmite (JCPDS, 00-076-1871, AlO(OH)). As reported by other authors [62,63], the boehmite formation was a result of the reaction between γ-Al_2_O_3_ and H_2_O. The boehmite crystallite size was between 15 and 30 nm (Table 7); adding Ca decreased its crystallite size. The Ni crystallite size was between 6 and 20 nm (Table 7). An increase in the calcination temperature and adding Ca to the catalysts increased this size. The large Ni crystallite sizes of catalysts calcined at 675 °C could be due to the high reduction temperature (700 °C). The boehmite and Ni crystallite sizes were calculated using the Scherrer equation for the main diffraction peak at 2θ = 14.4° and 44.5°, respectively. The main peak characteristic of NiAl_2_O_4_ at 2θ = 37.0° decreased with the increase in the calcination temperature and the addition of Ca, while the main peak for the γ-Al_2_O_3_ at 2θ = 45.7° also increased and could be related to the almost complete reduction of NiAl_2_O_4_, according to the H_2_-TPR results.

In addition, it was observed that the Ni/AlCa (Ni/AlCa-500 and Ni/AlCa-675) catalysts had an additional peak corresponding to the CaCO_3_ (JCPDS, 00-072-1937) phase after the APR of glycerol. The CaCO_3_ was formed by the reaction between CaO and CO_2_ [41] during the process.

The presence of SiO_2_ (JCPDS, 00-046-1045) in the catalysts after use was due to the incomplete separation between the catalyst and the inert sand before the characterization analysis.

The morphology of the catalysts changed after their use, and the boehmite morphologies in their structure were more clearly seen. These boehmite morphologies (Figure S2) were observed as thick plates, elongated shapes, and platelet-like particles [47,64], where they were not dependent on the calcination temperature or the addition of Ca. Koichumanova et al. [20] reported the results of boehmite as a catalyst support, showing phase stability and remarkably stable activity and improving the rate of H_2_ formation.

### 2.3. Results from Refined Crude Glycerol

**Figure 9:**
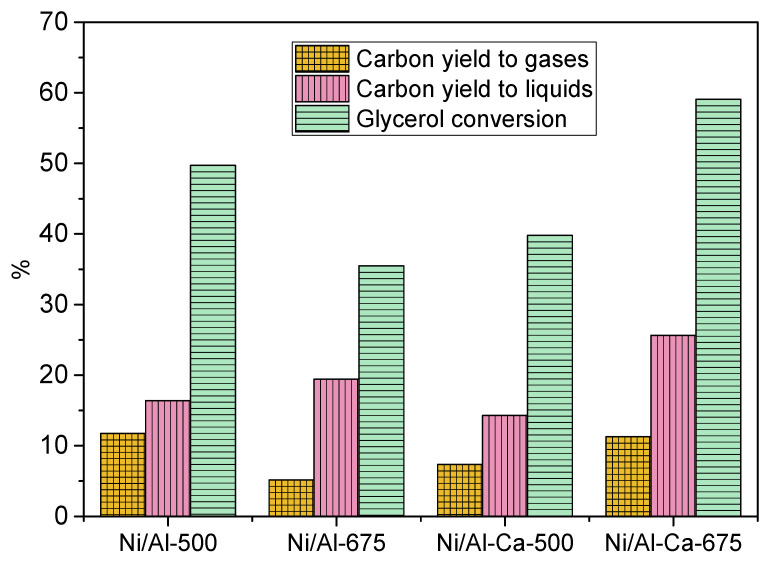
Catalytic performance using refined crude glycerol as feed.

Figure 9 displays the effect of the different catalysts when feeding refined crude glycerol during the APR reaction. The catalytic performance was not the same as when glycerol without impurities was used as the feed. When using refined crude glycerol, it was also observed that the Ni/AlCa-675 presented the best catalytic performance due to its high glycerol conversion and carbon yield to gases and liquids in comparison with the others. This means that adding Ca to the catalysts and increasing the calcination temperature favored the catalytic activity. Moreover, no influences were observed on the distribution of the carbon selectivity to liquid products as a function of the catalyst (Figure S3). The same trend was observed using pure glycerol.

The same trend in the composition of H_2_ with the pure glycerol was observed when using refined crude glycerol (Table 8). The calcination temperature and the addition of Ca favored an increase in the hydrogen content in the product gas and the H_2_/CO_2_ ratio in the APR of refined crude glycerol. The catalysts showed an H_2_/CO_2_ ratio of around 0.39 to 0.88, indicating that some hydrogen was consumed in parallel reactions.

**Figure 10:**
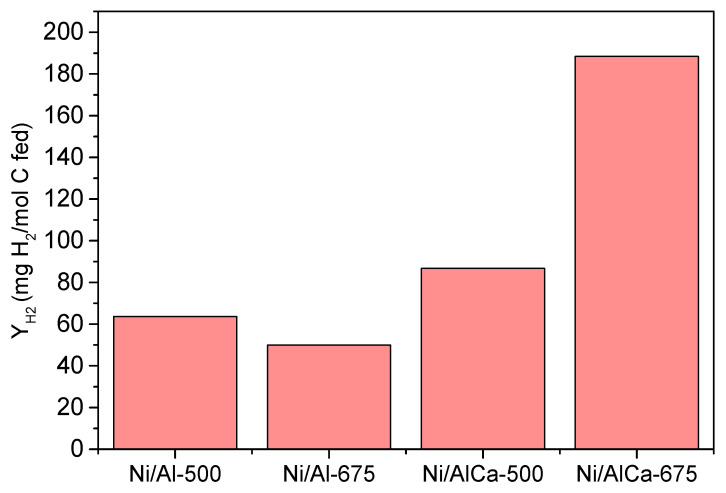
Hydrogen yield (Y_H2_) of all the catalysts during the APR of glycerol using refined crude glycerol as feed.

Furthermore, it was observed that adding Ca to the Ni/Al catalyst favored the hydrogen yield. The increase in the calcination temperature only benefitted the samples with Ca, the highest value being obtained with the Ni/AlCa-675 (Figure 10). Also, this was the catalyst with the highest catalytic activity. 

Using refined crude glycerol as the feed, the production of H_2_ follows the order Ni/AlCa-675 > Ni/AlCa-500 > Ni/Al-500 > Ni/Al-675. There is not a clear relationship between total basicity expressed as μmol CO_2_/g cat and the yield of H_2_. However, it is observed that the Ni/Al-675 catalyst showed the lowest H_2_ yield, the lowest total basicity (73.56 μmol CO_2_/g cat), and the lowest amount of relatively strong basic sites (14.6 μmol CO_2_/g cat).

After their use, the specific surface area, pore volume, and diameter of the catalysts decreased, as shown in Table 9. 

After use, all of the catalysts had phases corresponding to Ni, γ-Al_2_O_3_, NiAl_2_O_4_, and boehmite in their structure, which was the same as when pure glycerol was used as the feed (see Figure 8B). In this case, the boehmite crystallite size was between 15 and 40 nm (Table 9). Adding Ca decreased the crystallite size only with the catalyst calcined at 500 °C. The Ni crystallite size was between 7 and 20 nm (Table 9), with the size increasing when the calcination temperature was increased and Ca was added, which was the same trend as that observed when using glycerol without impurities. The main peak characteristic of NiAl_2_O_4_ at 2θ = 37.0° decreased with the increase in the calcination temperature and the addition of Ca, while the main peak for the γ-Al_2_O_3_ at 2θ = 45.7° increased. The catalysts with Ca (Ni/AlCa-500 and Ni/AlCa-675) presented an additional peak corresponding to the CaCO_3_ phase after the APR of refined crude glycerol. 

The morphology of the catalysts also changed after their use. The boehmite morphology in their structure was clearer. There was a significant difference in the Ni/AlCa-675 catalyst, which showed boehmite morphology with larger sizes in concordance with the XRD results (Figure S4).

### 2.4. Overall: Effect of the Type of Glycerol

The S_BET_ of the catalysts after use showed a different behavior depending on the glycerol and the samples. For the Ni/Al catalysts, the S_BET_ decreased after the APR of both pure and refined crude glycerol. For the Ni/AlCa catalysts, the S_BET_ increased after the APR of pure glycerol, while the S_BET_ diminished during the APR of refined crude glycerol. Reynoso et al. [42] found that the S_BET_ value also depended on the type of feed used. It decreased in the APR of the by-product (=refined crude glycerol) and increased during the APR of acetic acid. No change was observed in the APR of methanol. 

Regardless of the raw material, the catalysts presented the same structure (XRD results), and the stoichiometric H_2_/CO_2_ (range from 0.34 to 0.88) increased with an increase in the calcination temperature and addition of Ca. The H_2_ yield did not decrease with the Ni/Al-500 and Ni/AlCa-675 catalysts, but the Ni/Al-675 and Ni/AlCa-500 showed the worst H_2_ yield using refined crude glycerol. The combination of the presence of Ca and the high calcination temperature displayed promising results for the APR of refined crude glycerol. The acidity, basicity, and the metal content of the Al, Ni, and Ca are determinative for the catalytic performance of the catalysts.

Feeding both pure glycerol and refined crude glycerol into the reactor during APR over the catalysts with Ca enhanced the high H_2_ yield, achieving 145 and 188 mg H_2_/mol C fed, respectively, with the Ni/AlCa-675 catalyst. This means that using refined crude glycerol during APR over a Ni/AlCa-675 catalyst is a promising result from the point of view of improving the economic viability of biodiesel production.

## 3. Experimental Section

### 3.1. Crude Glycerol and Purification Process

The crude glycerol used for this work was obtained in our laboratory from the transesterification of sunflower oil with methanol as an alcohol, utilizing KOH as alkaline catalyst (oil/alcohol = 1/6 molar ratio; a mass of catalyst = 1% oil mass), following the procedure to obtain biodiesel described by García et al. [4]. 

On the whole, the crude glycerol (pH ≈ 12) was neutralized with concentrated acetic acid until a pH of around 6 was reached, followed by the evaporation and centrifugation/decantation methods until obtaining the purified glycerol (named “refined crude glycerol”). The excess of methanol was removed during the evaporation step using a rotary evaporator at 60 °C. It is a typical method in the industry to remove alcohol from both the biodiesel and the glycerol phase to use either a flash unit or an evaporator [65].

The most relevant physicochemical properties of the crude and refined crude glycerol are shown in Table 10, which shows the reduction in the amount of methanol after the purification process as well as an increase in the glycerol purity. 

### 3.2. Synthesis and Characterization of the Catalysts

The Ni/Al catalysts with 28 molar % of Ni (the molar ratio Ni/(Ni+Al)) were synthesized by the co-precipitation method, as reported in our previous work [47]. The Ni/AlCa catalysts were also prepared by the co-precipitation method, combining the procedure described in two studies [47,50], containing 28 molar % of Ni (the molar ratio Ni/(Ni+Al+Ca)) and a molar ratio of Ca/Al of 7.5%. Nickel nitrate [Ni(NO_3_)_2_·6H_2_O] (Sigma-Aldrich, purity: 97.0%, St. Louis, MO, USA), aluminum nitrate [Al(NO_3_)_3_·9H_2_O] (Fluka analytical, purity: 98.0%, St. Louis, MO, USA), and calcium nitrate [Ca(NO_3_)_2_·4H_2_O] (Sigma-Aldrich, purity: 99.0%, St. Louis, MO, USA) were used as metal precursors, while ammonium hydroxide (NH_4_OH) was employed as a precipitant. The mixture of nitrates was dissolved in milli-Q water and heated to 40 °C. Then, the NH_4_OH was added slowly with continuous vigorous stirring until the pH reached 7.8, and a gel was obtained. Subsequently, the catalyst-hydrated precursor was dried overnight at 70 °C in an oven.

All the samples (Ni/Al and Ni/AlCa catalysts) were calcined at 500 and 675 °C (heating rate: 1 °C/min) for 3 h in a furnace and sieved to a mesh size of 160–315 μm. 

The calcined and used catalysts were characterized by different techniques, including hydrogen temperature-programmed reduction (H_2_-TPR), inductively coupled plasma optical emission spectrometry (ICP-OES), X-ray diffraction (XRD), field emission scanning electron microscopy (FESEM), and N_2_-physisorption. The procedure for each technique was described in our previous work [47]. The acidity and basicity of the reduced catalysts were analyzed by temperature-programmed desorption of ammonia (NH_3_-TPD) and carbon dioxide (CO_2_-TPD), respectively.

The test of the NH_3_-TPD was analyzed in a Micromeritics AutoChem II 2920 instrument with a thermal conductivity detector (TCD) (Micromeritics, Norcross, GA, USA). The calcined samples were dried in an Ar stream at 120 °C for 1 h (heating rate 10 °C/min, total flow 50 mL/min) and then cooled at 45 °C. Prior to the analysis, the samples were activated in situ under a 10% H_2_/Ar for 1 h (heating rate 10 °C/min, total flow 50 mL/min) until 600 °C (for catalysts calcined at 500 °C) and 700 °C (for samples calcined at 675 °C) and then cooled at 100 °C with a He stream. The ammonia adsorption was carried out at 100 °C using a mixture of 5% NH_3_/He with a flow rate of 50 mL/min for 1 h. After the adsorption, the samples were purged with flowing He at 100 °C for 1 h to remove the physisorbed ammonia. The desorption of the chemisorbed ammonia was measured by heating the catalysts to 770 °C at a rate of 10 °C/min (total flow 30 mL/min) [47]. 

The analysis of the CO_2_-TPD was performed again in a Micromeritics AutoChem II 2920 instrument with a thermal conductivity detector (TCD) (Micromeritics, Norcross, Georgia, USA). First, the calcined samples were dried in an Ar stream at 120 °C for 1 h (heating rate 10 °C/min, total flow 50 mL/min) and then cooled at 45 °C. Before the analysis, the catalysts were activated in situ under a 10% H_2_/Ar for 1 h (heating rate 10 °C/min, total flow 50 mL/min) until 600 °C (for catalysts calcined at 500 °C) and 700 °C (for samples calcined at 675 °C) and then cooled at 80 °C with an Ar stream. The CO_2_ adsorption was carried out at 80 °C using a mixture of 5% CO_2_/Ar for 1 h with a 50 mL/min flow rate. After the adsorption, the samples were purged with flowing Ar at 80 °C for 1 h to remove the physisorbed CO_2_ and cooled at 45 °C with Ar. During the desorption analysis, the m/z = 44 signal was monitored in an Oministar GSD320 mass spectrometer. The temperature increased from 45 to 770 °C (heating rate of 10 °C/min, flow rate of 30 mL/min, Ar).

### 3.3. Catalytic Performance

The catalytic tests were performed in a small laboratory-scale continuous feeding unit designed and developed by PID (Process Integral Development Eng & Tech, Madrid, Spain), which mainly consisted of a stainless-steel fixed bed reactor (inner diameter = 9 mm) heated up with an electric furnace and a micrometric valve that regulates the pressure system. The figure of the experimental setup and more details can be found in our previous work [47]. The fixed bed is composed of a mixture of catalyst (2 g) and inert sand (5 g) with the same mesh size (160–315 µm) and is placed inside the tubular reactor between quartz wool supports. The catalysts were tested at 238 °C and 37 absolute bar for 3 h, using a solution of 5 wt.% of glycerol (pure or refined crude glycerol) with a liquid flow rate of 1 mL/min, obtaining gas and liquid products. Before the APR reaction, the samples were reduced in situ under 100 cm^3^ (STP)/min of H_2_ for 1 h at 600 °C (catalysts calcined at 500 °C) or 700 °C (samples calcined at 675 °C), according to the H_2_-TPR results. The exit gas mixture (H_2_, CH_4_, C_2_H_6_, CO_2_, CO, C_3_H_8_, and N_2_) was examined online employing an Agilent 490 Micro-GC equipped with Thermal Conductivity Detectors (TCD) (Santa Clara, CA, USA). The N_2_ was used as an internal standard. The liquid products (acetol, acetic acid, 1,2-propanediol (1,2-PDO), ethanol (EtOH), ethylene glycol (EG), methanol (MeOH), and non-reacted glycerol) were analyzed offline with an Agilent 7820A GC equipped with a Flame Ionization Detector (FID) and an HP-FFAP Agilent 19091F-105 capillary column (Santa Clara, CA, USA). The total C fed was analyzed offline using Total Organic Carbon (TOC) equipment (Shimadzu, Kyoto, Japan) to determine the hydrogen yield expressed as mg H_2_/mol C fed.

The catalytic performance was calculated using Equations (7)–(10) below.

The global glycerol conversion was calculated as follows:

(7) $$Glycerol\;conversion\;\left(\%\right)=\frac{n_{glycerol}^{in}-n_{glycerol}^{out}}{n_{glycerol}^{in}}\times 100$$

where $n_{glycerol}^{in}$ and $n_{glycerol}^{out}$ are the moles of glycerol fed and the unreacted glycerol in the exit liquid, respectively. 

The carbon yield to liquids and the carbon yield to gases were defined as follows:

(8) $$Carbon\;yield\;to\;liquids\;\left(\%\right)=\frac{n_{MeOH}+2n_{EtOH}+2n_{Acetic\;acid}+3n_{Acetol}+3n_{1,2-PDO}+2n_{EG}}{\mathrm{Total}\;C\;\mathrm{moles}\;\mathrm{in}\;\mathrm{the}\;\mathrm{feedstock}}\times 100$$

(9) $$Carbon\;yield\;to\;gases\;\left(\%\right)=\frac{n_{CO}+n_{CO_{2}}+n_{CH_{4}}+2n_{C_{2}H_{6}}+3n_{c_{3}H_{8}}}{\mathrm{Total}\;C\;\mathrm{moles}\;\mathrm{in}\;\mathrm{the}\;\mathrm{feedstock}}\times 100$$

where $n_{i}$ are the moles of the *i* product (*i* = each liquid or gas product).

The H_2_ yield was calculated as follows:

(10) $$Hydrogen\;yield\;\left(\frac{\mathrm{mg}\;H_{2}}{\mathrm{mol}\;C\;\mathrm{fed}}\right)=\frac{\;n_{H_{2}}\times MW_{H_{2}}\times 1000}{\mathrm{Total}\;C\;\mathrm{moles}\;\mathrm{in}\;\mathrm{the}\;\mathrm{feedstock}}$$

where $n_{H_{2}}$ are the moles of H_2_ and $MW_{H_{2}}$ is the molecular weight of the H_2_ (2 g/mol). 

The total C moles in the feedstock were analyzed by the TOC analysis.

The carbon selectivity to liquid products was defined as the percentage ratio of carbon in a liquid product to the total carbon in all the liquid products analyzed where unreacted glycerol was not considered.

Due to errors in analyzing and collecting the samples, there is a slight difference between the glycerol conversion and the addition of the carbon yield to products (gas and liquid). As reported by other authors, an experiment with a carbon deficit below 15% was considered a reliable test [33,66]. The carbon deficit (using the pure glycerol as feed) was defined as follows:

(11) Carbon deficit=Glycerol conversion−(carbon yield to gases+carbon yield to liquids)

## 4. Conclusions

Ni/Al and Ni/AlCa catalysts were prepared using the co-precipitation method, calcined at different temperatures (500 and 675 °C), and characterized by several techniques. Their performance in the APR of glycerol was analyzed at 238 °C and 37 absolute bar, using a solution of 5 wt.% of glycerol (with and without impurities) in milli-Q water.

The increase in the calcination temperature decreased the surface area, acidity, and basicity but increased the crystallinity of the fresh catalysts. After the APR reaction, the Ni crystallite size increased more for the samples calcined at a high calcination temperature (675 °C), probably due to the high activation temperature required (700 °C) that caused sintering. Moreover, the high calcination temperature positively influenced the H_2_ yield using pure glycerol as feed, but only for the Ca-containing catalyst when using refined crude glycerol.

Adding Ca to the catalyst diminished the surface area and acidity but increased the basicity of the fresh samples. In addition, the proportion of NiO phases with low interaction with the support was increased. Regardless of the feedstock, the H_2_ yield increased with the Ca-containing catalysts.

Depending on the raw material, the catalytic performance changed. The Ni/Al-675 and Ni/AlCa-500 catalysts produced a lower H_2_ yield using refined crude glycerol than pure glycerol. A similar H_2_ yield was obtained with the Ni/Al-500 catalyst irrespective of the feed type, while for the Ni/AlCa-675 catalyst, the highest H_2_ yield was observed using refined crude glycerol.

Overall, the incorporation of Ca into the Ni/Al catalysts (Ni/AlCa) and the increase in the calcination temperature of the Ni/AlCa catalysts, regardless of the feedstock, favored the production of H_2_. The highest value H_2_ yield (188 mg H_2_/mol C fed) was obtained during the APR of refined crude glycerol over the Ni/AlCa-675 catalyst. This means that using refined crude glycerol during the APR over the Ni/AlCa-675 catalyst is a promising route towards improving the economic viability of biodiesel production.

## Figures and Tables

**Table 1:** Composition of the calcined catalysts.

Sample	Ni ^1^ (molar%)	Al ^1^ (molar%)	Ca ^1^ (molar%)	Ni ^2^ (molar%)	Al ^2^ (molar%)	Ca ^2^ (molar%)	Ca/Al ^2^ (molar%)
Ni/Al-500	28	72	-	28.3 ^3^	71.7 ^3^	-	0
Ni/Al-675	28	72	-	29.0	71.0	-	0
Ni/AlCa-500	28	67	5	25.9	69.1	5.0	7.2
Ni/AlCa-675	28	67	5	28.5	66.4	5.1	7.7

^1^ Theorical value. ^2^ Analysis value determined by ICP-OES. ^3^ Raso et al. [47].

**Table 2:** Textural properties of the calcined catalysts.

Sample	S_BET_ ^1^ (m^2^/g)	v_p_ ^2^ (cm^3^/g)	d_p_ ^2^ (nm)
Ni/Al-500	256	0.145	3.7
Ni/Al-675	203	0.193	3.9
Ni/AlCa-500	232	0.147	3.3
Ni/AlCa-675	188	0.180	4.4

^1^ The BET method. ^2^ The BJH adsorption method.

**Table 3:** H_2_-TPR results of the calcined samples.

Sample	Temperature (°C)/Relative Amount (%) ^1^		Total H_2_ Consumption Ni_red_ ^2^	
	T1/F1	T2/F2	mmol H_2_/g_cat_	%
Ni/Al-500	343/2.1	633/97.9	4.42	90.21
Ni/Al-675	-	708/100.0	4.31	86.01
Ni/AlCa-500	329/8.8	613/91.2	4.78	100
Ni/AlCa-675	418/8.0	733/92.0	4.40	89.41

^1^ Calculated from Gaussian deconvolution of H_2_-TPR profiles. ^2^ Reduced Ni.

**Table 4:** NH_3_-TPD results of the reduced samples.

Sample	Temperature (°C)/Relative Amount (%) ^1^				Total NH_3_ Desorption	
	T1/F1	T2/F2	T3/F3	T4/F4	μmol NH_3_/g_cat_	μmol NH_3_/m^2^
Ni/Al-500	170/7.3	274/37.1	503/55.6	-	919.58	3.59
Ni/Al-675	170/10.2	275/41.9	480/47.9	-	548.75	2.70
Ni/AlCa-500	184/9.0	253/28.1	455/47.7	703/15.2	531.93	2.29
Ni/AlCa-675	182/8.3	265/20.4	461/47.6	711/23.7	493.25	2.62

^1^ Calculated from Gaussian deconvolution of NH_3_-TPD profiles.

**Table 5:** CO_2_-TPD results of the reduced samples.

Sample	Temperature (°C)/Relative Amount (%) ^1^			Total CO_2_ Desorption	
	T1/F1	T2/F2	T3/F3	μmol CO_2_/g_cat_	μmol CO_2_/m^2^
Ni/Al-500	108/13.3	170/53.4	226/33.3	81.44	0.32
Ni/Al-675	110/9.4	176/70.7	243/19.9	73.56	0.46
Ni/AlCa-500	115/14.8	168/38.6	242/46.6	156.17	0.67
Ni/AlCa-675	114/14.1	159/34.3	235/51.6	118.52	0.63

^1^ Calculated from Gaussian deconvolution of CO_2_-TPD profiles.

**Table 6:** Gas composition (vol%, N_2_, and H_2_O free), using pure glycerol as feed.

Sample	H_2_	Others ^1^	H_2_/CO_2_
Ni/Al-500	16.8	83.2	0.34
Ni/Al-675	29.9	70.1	0.53
Ni/AlCa-500	39.7	60.3	0.68
Ni/AlCa-675	36.8	63.2	0.73

^1^ Others: CO_2_, CH_4_, C_2_H_6_, and C_3_H_8_.

**Table 7:** Textural properties, boehmite, and Ni crystallite sizes of the used catalyst.

Sample	S_BET_ ^1^ (m^2^/g)	v_p_ ^2^ (cm^3^/g)	d_p_ ^2^ (nm)	D_boehmite_ ^3^ (nm)	D_Ni_ ^3^ (nm)
Ni/Al-500	241 (±8)	0.089 (±0.020)	3.1 (±0.0)	28.3	5.8
Ni/Al-675	134 (±3)	0.168 (±0.004)	6.8 (±0.4)	29.9	19.9
Ni/AlCa-500	244 (±4)	0.097 (±0.008)	3.0 (±0.0)	20.0	14.4
Ni/AlCa-675	192 (±8)	0.132 (±0.012)	3.1 (±0.1)	15.0	20.4

^1^ The BET method. ^2^ The BJH adsorption method. ^3^ Boehmite and Ni crystallite sizes calculated from the Scherrer equation.

**Table 8:** Gas composition (vol%, N_2_, and H_2_O free) using refined crude glycerol as feed.

Sample	H_2_	Others ^1^	H_2_/CO_2_
Ni/Al-500	21.8	78.2	0.39
Ni/Al-675	33.3	66.8	0.51
Ni/AlCa-500	37.8	62.2	0.64
Ni/AlCa-675	46.1	53.9	0.88

^1^ Others: CO_2_, CH_4_, C_2_H_6_, and C_3_H_8_.

**Table 9:** Textural properties of the used catalysts using refined crude glycerol as feed.

Sample	S_BET_ ^1^ (m^2^/g)	v_p_ ^2^ (cm^3^/g)	d_p_ ^2^ (nm)	D_boehmite_ ^3^ (nm)	D_Ni_ ^3^ (nm)
Ni/Al-500	235	0.108	3.0	23.0	6.7
Ni/Al-675	176	0.128	3.3	24.3	19.5
Ni/AlCa-500	216	0.132	3.1	15.0	14.5
Ni/AlCa-675	154	0.152	4.2	39.6	19.1

^1^ The BET method. ^2^ The BJH adsorption method. ^3^ Boehmite and Ni crystallite sizes calculated from the Scherrer equation.

**Table 10:** Physicochemical properties of crude and refined crude glycerol.

	Crude Glycerol	Refined Crude Glycerol
Physical properties		
pH	11.87 ± 0.05	5.77 ± 0.15
Density (g/cm^3^)	1.096 ± 0.000	1.265 ± 0.001
LHV (MJ/kg)	22.673 ± 0.006	16.604 ± 0.00
Chemical composition		
Glycerol (wt.%)	61.69 ± 5.56	82.32 ± 4.34
Methanol (wt.%)	25.91 ± 4.86	1.17 ± 0.52
Acetic acid (wt.%)	0	3.15 ± 0.70
Elemental analysis		
C (%)	31.19 ± 0.25	26.60 ± 0.40
H (%)	6.77 ± 0.15	6.24 ± 0.20
O * (%)	62.05 ± 0.39	67.16 ± 0.59

* Determined by difference.

## Data Availability

Most of data are available in this manuscript. More specific data can be requested to the authors.

## Supplementary Materials

The following supporting information can be downloaded at: https://www.mdpi.com/article/10.3390/molecules28186695/s1, Figure S1: Carbon selectivity to liquid products of the catalysts during APR of pure glycerol; Figure S2: FESEM images of the used catalysts using pure glycerol as feed; Figure S3: Carbon selectivity to liquid products of the catalysts during APR of refined crude glycerol; Figure S4: FESEM images of the used catalysts using refined crude glycerol as feed.
