# Supplementary material for: Renewable Hydrogen Production by Aqueous Phase Reforming of Pure/Refined Crude Glycerol over Ni/Al-Ca Catalysts
Source: Molecules. 2023 Sep 19;28(18):6695. doi: 10.3390/molecules28186695 (PMC10536820; doi:10.3390/molecules28186695)
Supplement: Supplementary file 1 [file molecules-28-06695-s001.zip › molecules-2591928-supplementary.pdf]

Renewable Hydrogen Production by Aqueous Phase Reforming of Pure/Refined Crude Glycerol over Ni/Al-Ca Catalysts

Raquel Raso, Eduardo Abad, Lucía García *, Joaquín Ruiz, Miriam Oliva and Jesús Arauzo

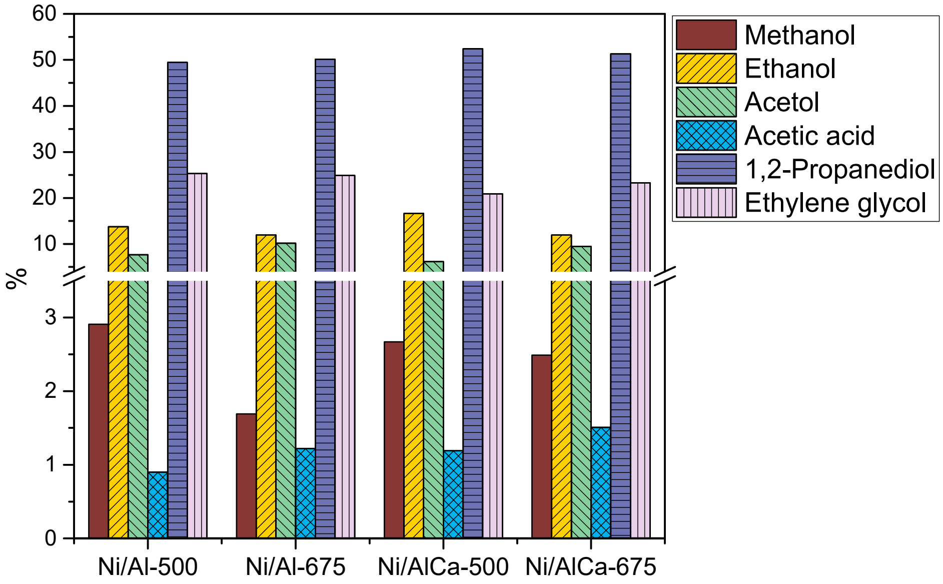

Figure S1. Carbon selectivity to liquid products of the catalysts during APR of pure glycerol.

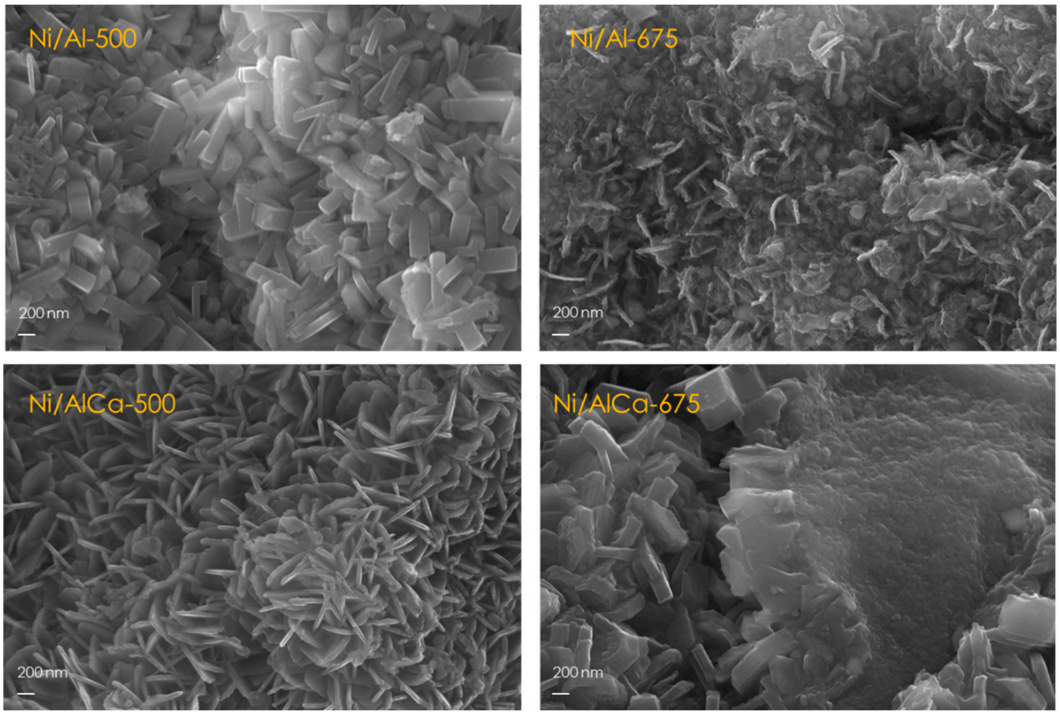

Figure S2. FESEM images of the used catalysts using pure glycerol as feed.

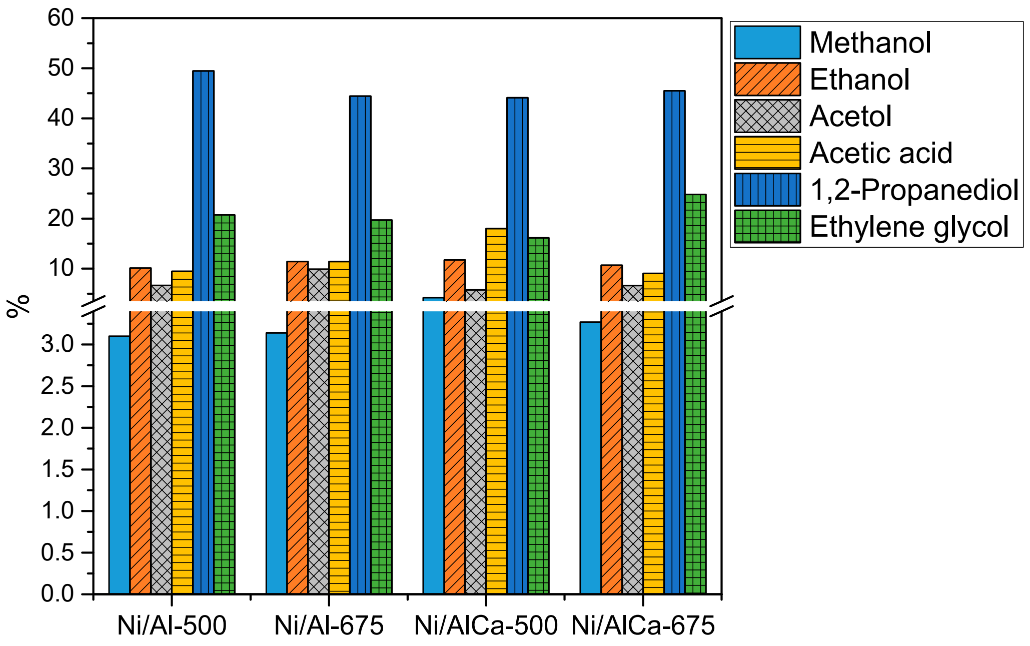

Figure S3. Carbon selectivity to liquid products of the catalysts during APR of refined crude glycerol.

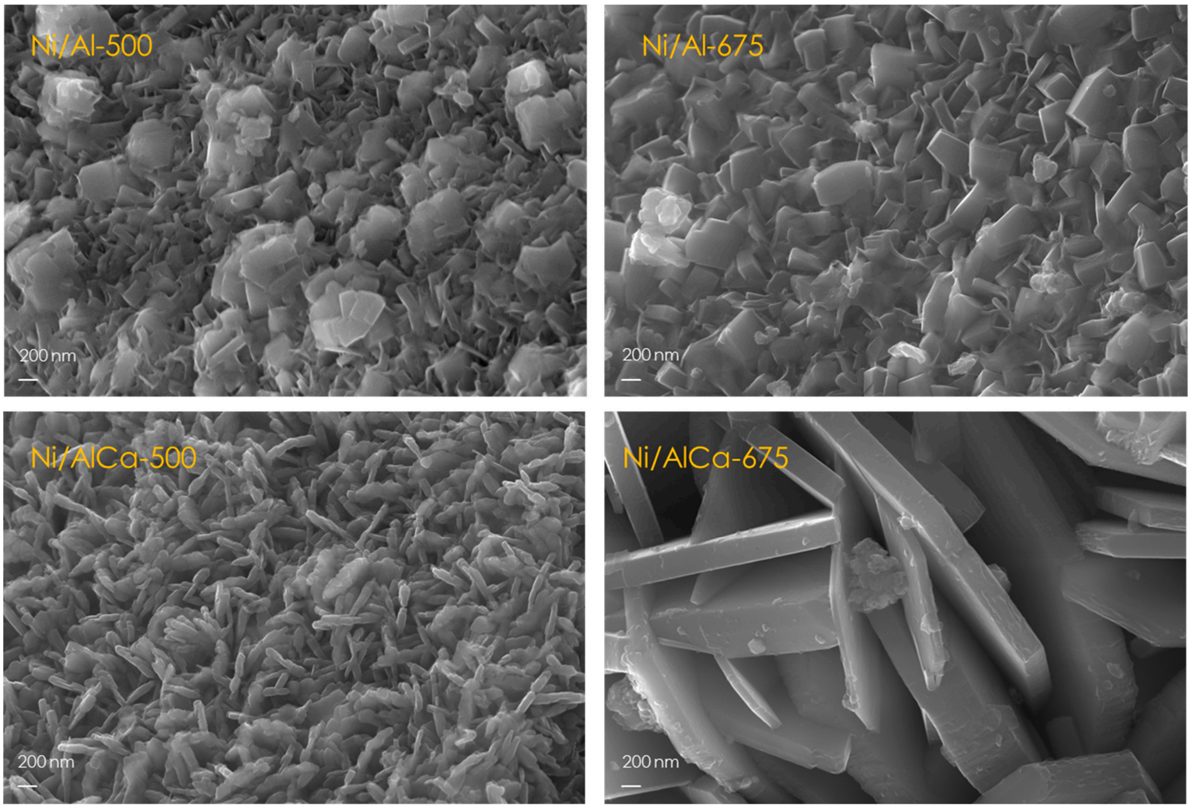

Figure S4. FESEM images of the used catalysts using refined crude glycerol as feed.
